# Biphasic Calcium Phosphate Versus Deproteinized Bovine Bone Mineral for Sinus Floor Elevation: A Systematic Review and Meta-Analysis of Randomized Controlled Trials

**DOI:** 10.7759/cureus.87573

**Published:** 2025-07-09

**Authors:** Mohammed Alkandari, Meshari Alkandari, Rashed Alhallaq, Mohammad Mohammad, Daniah Safar, Abdullah Alobaidan, Mubarak Alobaidan, Sultan Albusarah, Awrad Enki, Jumanah Malallah, Abdulwahab T Alenezi

**Affiliations:** 1 Department of Dentistry, Rumaithiya Polyclinic, Kuwait City, KWT; 2 Department of Dentistry, West Salmiya Polyclinic, Kuwait City, KWT; 3 Department of Dentistry, Al-Adan Specialized Health Center, Kuwait City, KWT; 4 Department of Dentistry, Al-Qurain Polyclinic, Mubarak Al-Kabeer, KWT; 5 Department of Dentistry, Saad Al-Abdullah Health Center Block 2, Al Jahra, KWT

**Keywords:** biphasic calcium phosphate, deproteinized bovine bone mineral, histomorphometry, meta-analysis, sinus floor elevation

## Abstract

Sinus floor elevation using deproteinized bovine bone mineral (DBBM) is widely used as a grafting material to promote new bone formation. Biphasic calcium phosphate (BCP) has also been utilized to enhance vertical and horizontal bone augmentation prior to dental implant placement. Both materials are considered viable options for sinus augmentation. This systematic review and meta-analysis aimed to evaluate the histological results of BCP compared to DBBM. A systematic search of PubMed, Scopus, Web of Science, and Cochrane Controlled Register of Trials (CENTRAL) was conducted from March 2025 to May 2025 to identify randomized controlled trials (RCTs) comparing BCP versus DBBM in patients undergoing sinus floor elevation. Primary outcomes included the percentage of new bone formation and residual bone graft. The secondary outcome was the percentage change in soft tissue. Pooled mean differences (MD) with 95% confidence intervals (CI) were calculated using a random-effects model. All analyses were performed using Stata/MP Version 18 (StataCorp LLC, College Station, Texas, United States). Ten RCTs involving 328 patients and 389 implants were included. BCP was associated with a higher percentage of new bone formation (MD=3.48; 95% CI: 0.89-5.97; p=0.01; I^2^=44.03), lower residual grafting materials (MD=-8.41; 95% CI: -13.12 to -3.72; p<0.001; I^2^=85.71), and higher soft tissue materials (MD=6.01; 95% CI: 2.39-9.63; p<0.001; I^2^=71.96) compared to DBBM. In conclusion, the adjunctive use of BCP enhances histomorphometric outcomes in sinus floor elevation. Further high-quality, long-term RCTs with standardized protocols are needed to confirm these findings.

## Introduction and background

Maxillary sinus pneumatization and bone resorption after long periods of maxillary teeth loss can lead to a significant reduction in the available bone for implants. Moreover, dental implanting requires specific bone regenerative measures and adequate vertical bone height [1,2]. This anatomical limitation often delays the immediate placement of dental implants without prior bone augmentation [3]. Sinus floor elevation has become the go-to procedure with reliable long-term efficacy measurements to create a suitable environment for implants [4]. This procedure involves elevating the Schneiderian membrane, which lines the maxillary sinus, and then filling it with a bone graft material, aiming to allow new bone formation and increase the vertical bone height [5].

Deproteinized bovine bone mineral (DBBM), commonly referred to as Bio-Oss, is a well-documented xenograft extensively studied in clinical trials [2]. It functions primarily as an osteoconductive scaffold due to its porous structure and slow resorption rate, facilitating new bone formation and volume stability [1]. While DBBM is considered highly osteoconductive, its osteoinductive property remains limited. Although some studies suggest that DBBM may exhibit minimal osteoinductive potential, it is generally regarded as a passive scaffold lacking intrinsic osteogenic properties [2,5].

In addition to xenografts, there has been significant advancement in synthetic bone grafts, particularly with the emergence of biphasic calcium phosphates (BCPs) as a prominent alternative [5-7]. BCP grafts are composed of hydroxyapatite (HA) and tribasic calcium phosphate (TCP) with different ratios, 60% HA and 40% TCP being the most common [1,8]. The idea of BCP grafts is to combine the osteoconductive scaffold properties and long-term stability offered by HA and the nature of TCP that helps with new bone formation by releasing calcium and phosphate ions while being resorbed [8]. Also, as was highlighted in previous clinical trials [1,3,5], the bioactivity and resorption rate of the BCPs can be tailored by modifications of the ratio between HA and TCP as well as other factors.

Despite the widespread use and published benefits of both DBBM and BCPs in sinus floor elevation procedures, a definitive conclusion regarding the superior material for optimal new bone formation and graft stability remains uncertain. Individual clinical trials and observational studies often present conflicting results [1,2,5,9]. Thus, we aimed to conduct this systematic review and meta-analysis on these two interventions to provide the clinical practice with better graft material in terms of new bone formation, graft residual, and other related outcomes.

## Review

Materials and methods

We followed the Preferred Reporting Items for Systematic Reviews and Meta-Analyses (PRISMA) guidelines [10] while conducting this systematic review and meta-analysis. Additionally, we adhered to the guidelines of the Cochrane Handbook for Systematic Reviews of Interventions [11].

Literature Search

A systematic search was conducted in PubMed, Web of Science (WOS), Scopus, and Cochrane Controlled Register of Trials (CENTRAL) from inception until May 2025. The search strategy included the following terms: ("Maxillary Sinus Floor Augmentation" OR "sinus lift" OR "sinus graft") AND ("biphasic calcium phosphate" OR "BCP" OR " Hydroxyapatites" OR " synthetic bone graft ") AND ("deproteinized bovine bone" OR "xenograft" OR "DBBM" OR "Bio-Oss"). The detailed search strategies for each database are presented in Table 1. We included only studies published in English. To ensure the comprehensiveness of the review, we also manually screened the reference lists of all included studies for additional relevant publications.

**Table 1:** Detailed search strategy for each database WOS: Web of Science; CENTRAL: Cochrane Controlled Register of Trials

Database	Search terms	Search field	Search results
PubMed	(((Maxillary Sinus Floor Augmentation) OR (sinus lift) OR (sinus graft)) AND ((biphasic calcium phosphate) OR (BCP) OR (Hydroxyapatites) OR (synthetic bone graft)) AND ((xenograft) OR (deproteinized bovine bone) OR (Bio-Oss) OR (DBBM)))	Title and abstract, English	132
CENTRAL	(((Maxillary Sinus Floor Augmentation) OR (sinus lift) OR (sinus graft)) AND ((biphasic calcium phosphate) OR (BCP) OR (Hydroxyapatites) OR (synthetic bone graft)) AND ((xenograft) OR (deproteinized bovine bone) OR (Bio-Oss) OR (DBBM)))	All fields, English	42
WOS	(((Maxillary Sinus Floor Augmentation) OR (sinus lift) OR (sinus graft)) AND ((biphasic calcium phosphate) OR (BCP) OR (Hydroxyapatites) OR (synthetic bone graft)) AND ((xenograft) OR (deproteinized bovine bone) OR (Bio-Oss) OR (DBBM)))	All fields, English	122
Scopus	(((Maxillary Sinus Floor Augmentation) OR (sinus lift) OR (sinus graft)) AND ((biphasic calcium phosphate) OR (BCP) OR (Hydroxyapatites) OR (synthetic bone graft)) AND ((xenograft) OR (deproteinized bovine bone) OR (Bio-Oss) OR (DBBM)))	Title, abstract, keywords, English	146

Eligibility Criteria

The selection of studies was carried out in two stages. Initially, all retrieved records were screened based on their titles and abstracts. Those deemed potentially relevant were then subjected to a full-text review to assess eligibility according to predefined criteria. Studies were included if they involved patients undergoing sinus floor elevations, with the intervention group receiving BCP and the comparison group receiving DBBM. Only studies that reported relevant outcomes using an intention-to-treat analysis were considered. We excluded studies that did not examine the BCP as their synthetic bone graft, as well as those with unpublished data, conference abstracts, or observational designs.

Outcomes

The primary outcome of interest was the percentage change of the new bone formation, with a higher percentage of bone formation indicating a favorable outcome [12]. Additionally, other studied outcomes were the percentage of residual bone, with a lower percentage indicating a favorable outcome [12], and the percentage of soft tissue calculated as the amount of fibrous tissue in the maxillary sinus cavity.

Quality Assessment

Two authors independently assessed the risk of bias of all included studies using the Cochrane Risk of Bias 2 (ROB-2) tool for evaluating randomized controlled trials (RCTs) [13]. The tool uses five different domains accounting for different methodologies of bias, which include selection bias (randomization process), performance bias (deviation from intended interventions), detection bias (outcome measurements), attrition bias (missing outcome data), and reporting bias (selection of the reported results). The decisions were labelled as "high risk of bias", "some concerns", and "low risk of bias". Any disagreements between the two authors were resolved via discussion with another author.

Data Extraction and Meta-Analysis

Data were extracted from the included studies using a standardized Excel spreadsheet (Microsoft Corporation, Redmond, Washington, United States). Extracted information was categorized into four key domains: (1) general study characteristics such as study design, country of origin, follow-up duration, reported outcomes, dental implant manufacturer, and number of sinuses; (2) patient-related data, including sample size per group, mean age, male proportion, residual bone height, and type of sinus lift approach; (3) risk of bias assessments; and (4) outcome measurements. Any disagreements between the authors were resolved with a third author. For continuous variables, we collected the mean and standard deviation (SD) as reported in each study at the final follow-up. Meta-analysis of continuous outcomes was performed using the mean difference (MD) with a 95% confidence interval (CI), applying the DerSimonian and Laird random-effects model. Statistical heterogeneity was evaluated using the Cochrane Q test and the I² statistic, with a p-value of <0.05 and I² of ≥50% indicating significant heterogeneity. Data synthesis was conducted in Stata/MP Version 18 (StataCorp LLC, College Station, Texas, United States) using the "meta esize" and "meta forest plot" packages.

Results

Search Results

**Figure 1:**
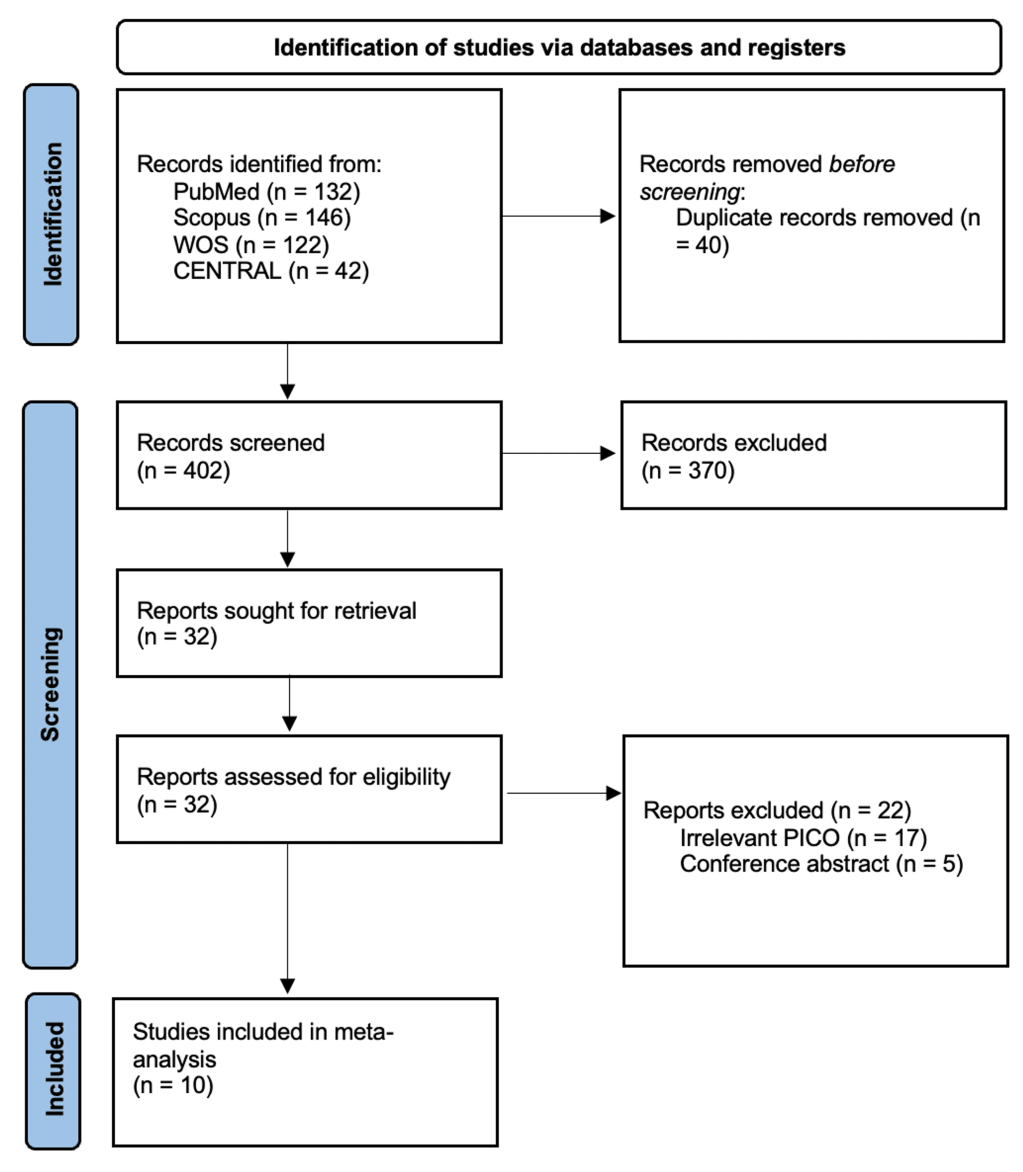
PRISMA flowchart PRISMA: Preferred Reporting Items for Systematic Reviews and Meta-Analyses; WOS: Web of Science; CENTRAL: Cochrane Controlled Register of Trials; PICO: population, intervention, comparison, and outcome

We identified 442 citations from databases, of which 40 articles were excluded following the removal of duplicates and finally 10 RCTs [1-9,14] were included in the final analysis following title, abstract, and full-text screening. The study selection process is summarized in the PRISMA chart (Figure 1).

Characteristics and Quality Assessment of the Included Studies

Ten RCTs comprising 328 patients and 389 were included, of which 161 patients (49.1%) were allocated to BCP, while 167 patients (50.9%) were allocated to DBBM. The mean age of the patients was 56.4±10.2 years, with 108 (43.2%) of the patients being females. Detailed baseline and summary characteristics of the included studies and patients are summarized in Table 1 and Table 2.

**Table 2:** Summary characteristics of the included studies BCP: biphasic calcium phosphate; DBBM: deproteinized bovine bone mineral; RFA: resonance frequency analysis; CT: computed tomography; CBCT: cone beam computed tomography; BV/TV ratio: bone volume/total volume ratio; GV/TV ratio: graft volume/total volume ratio; NB: new bone; RG: residual graft; ST: soft tissue; NR: not reported; DPBM: demineralized porcine bone matrix; MCBA: modified collagen-based allograft; AB: autologous bone

Study ID	Study design	Sample size	Number of implants	Intervention	Control	Outcomes	Dental implant manufacturer	Follow-up
Arunjaroensuk et al., 2024 [1]	Randomized controlled trial	24	24	BCP	DBBM (Bio-Oss)	Gene expression, micro-CT analysis (BV/TV ratio, GV/TV ratio), histomorphometrical analysis (new bone formation, residual graft area)	Straumann AG (Bone Level Tapered (BLT)) (Basel, Switzerland)	6 months
Cordaro et al., 2008 [6]	Randomized controlled trial	32	64	BCP	DBBM (Bio-Oss)	Histomorphometry (NB, RG, ST), bone-graft contact	Institut Straumann AG (SLActive® surface) (Basel, Switzerland)	6-8 months
de Lange et al., 2014 [14]	Randomized controlled trial	14	14	BCP	DBBM (Bio-Oss)	Micro-CT, histomorphometry	Camlog® screw line (Camlog Biotechnologies AG, Germany)	Up to 4 years
Froum et al., 2008 [7]	Randomized controlled trial	21	21	BCP	DBBM (Bio-Oss)	Vital bone content, connective tissue content, residual graft material content	NR	6-8 months
Oh et al., 2019 [6]	Randomized controlled trial	60	60	BCP	DBBM (Bio-Oss)	RFA, micro-CT, histomorphometric analysis	NR	21 months
Kraus et al., 2020 [2]	Randomized controlled trial	62	62	BCP	DBBM (Bio-Oss)	Histomorphometry (NB, RG, ST)	Straumann® dental implant system (SLActive®) (Basel, Switzerland)	6 months
Lindgren et al., 2012 [8]	Randomized controlled trial	22	22	BCP	DBBM (Bio-Oss)	Implant survival, radiographic and clinical parameters	Straumann® SLActive® surface implants (Basel, Switzerland)	1 year
Schmitt et al., 2024 [9]	Randomized controlled trial	17	17	BCP	DBBM (Bio-Oss), DPBM	Graft volume alterations (CBCT), histological characteristics	Implants planned; manufacturer not specified for 3D assessment phase	4-6 months
Schmitt et al., 2013 [3]	Randomized controlled trial	29	58	BCP	DBBM (Bio-Oss), MCBA, AB	Histomorphometry (NB, RG), bone volume	NR	5 months
Mordenfeld et al., 2015 [4]	Randomized controlled trial	47	47	BCP	DBBM (Bio-Oss), DPBM	Implant survival, radiographic and clinical parameters	Straumann® SLActive® surface implants (Basel, Switzerland)	5 years

**Table 3:** Patient's characteristics of the included studies BCP: biphasic calcium phosphate; DBBM: deproteinized bovine bone mineral

Study ID	Arms	Sample size	Age (years)		Sex (male, n, %)	Sinus lift approach	Graft manufacturer
			Mean	SD			
Arunjaroensuk et al., 2024 [1]	BCP	12	55.75	10.9	7 (58.3%)	Lateral window	DenaOss-M (70/30 HA/β-TCP, National Science and Technology Development Agency, Thailand)
	DBBM	12	55.67	10.85	9 (75%)		Cerabone® (Botiss Biomaterials GmbH, Germany)
Cordaro et al., 2008 [6]	BCP	14	NA		Not reported	Lateral sinus augmentation	Straumann® BoneCeramic (60/40 HA/β-TCP, Institut Straumann AG, Switzerland)
	DBBM	18					Bio-Oss® (Geistlich AG, Switzerland)
de Lange et al., 2014 [14]	BCP	8	66	NA	1 (20%)	Lateral window sinus elevation	Straumann® BoneCeramic (60/40 HA/β-TCP, Institut Straumann AG, Switzerland)
	DBBM	6					Bio-Oss® (Geistlich AG, Switzerland)
Froum et al., 2008 [7]	BCP	10	NA		NA	Lateral approach	Straumann® BoneCeramic (assumed 60/40 HA/β-TCP, Institut Straumann AG, Switzerland)
	DBBM	11					Bio-Oss® (Osteohealth) (Geistlich Pharma AG, Switzerland)
Oh et al., 2019 [5]	BCP	31	54.3	NA	33 (58.9%)	Sinus elevation (lateral approach)	Osteon™III (60/40 HA/β-TCP, Genoss, South Korea)
	DBBM	29	54.3	NA			Bio-Oss® (Geistlich, Switzerland)
Kraus et al., 2020 [2]	BCP	29	59.3	10.8	34%	Lateral window	Straumann® Vivoss™ (10/90 HA/TCP, Institut Straumann AG, Switzerland)
	DBBM	33					Bio-Oss® (Geistlich, Switzerland)
Lindgren et al., 2012 [8]	BCP	11	67	NA	5 (45.5%)	Lateral approach	BoneCeramic® (60/40 HA/TCP, Straumann®, Switzerland)
	DBBM	11					Bio-Oss® (Geistlich Biomaterials, Switzerland)
Schmitt et al., 2024 [9]	BCP	8	57	11.02	9 (60%)	Two-staged external sinus grafting	OSOPÍA® (>90% TCP/<10% HA, Regedent GmbH, Germany)
	DBBM	9	48.05	13.52			Geistlich Bio-Oss® (Geistlich Biomaterials GmbH, Germany)
Schmitt et al., 2013 [3]	BCP	14	NA		13 (43.3%)	Lateral window	Straumann® BoneCeramic (assumed 60/40 HA/TCP, Institut Straumann AG, Switzerland)
	DBBM	15					Geistlich Bio-Oss® (Geistlich Biomaterials GmbH, Germany)
Mordenfeld et al., 2015 [4]	BCP	24	67	NA	5 (45.5%)	Lateral window sinus elevation	BoneCeramic® (60/40 HA/TCP, Straumann®, Switzerland)
	DBBM	23					Bio-Oss® (Geistlich Biomaterials, Switzerland)

**Figure 2:**
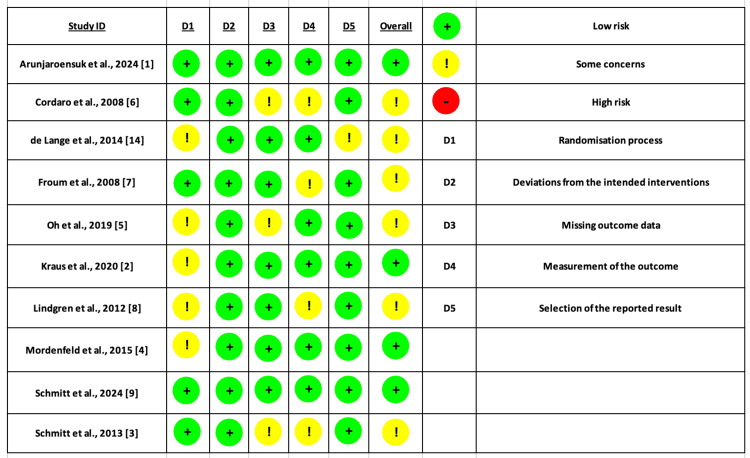
Risk of bias assessment using ROB-2 tool for RCTs ROB-2: Cochrane Risk of Bias 2; RCTs: randomized controlled trials

Regarding the risk of bias assessment using the ROB-2 tool, eight studies showed some concerns mainly due to the randomization process and missing data. Only two studies had an overall low risk of bias (Figure 2).

Outcomes

**Figure 3:**
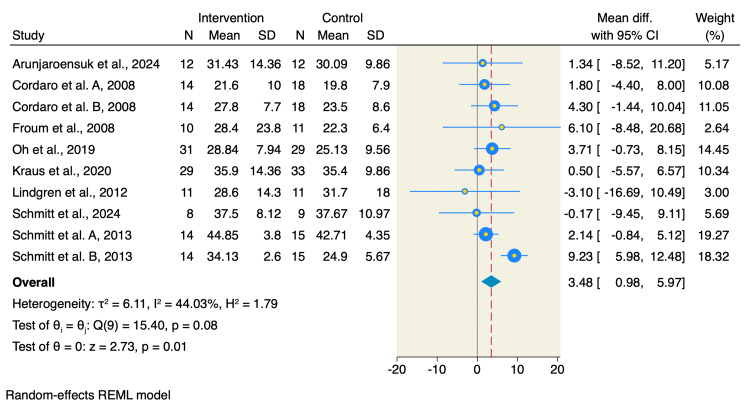
Forest plot of the percentage of new bone formation References: [1-9,14] REML: restricted maximum likelihood

Eight studies assessed the percentage of the new bone formation, of which BCP resulted in an increase of newly formed bone compared to DBBM (MD=3.48; 95% CI: 0.98-5.97; p=0.01; I^2^=44.03%; p=0.08) as shown in Figure 3.

**Figure 4:**
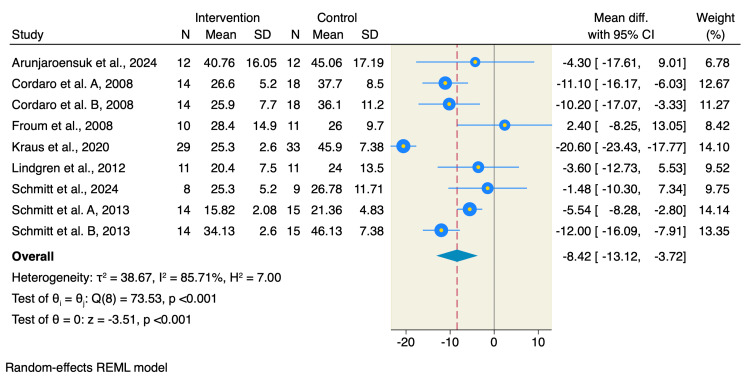
Forest plot of the percentage of residual bone graft References: [1-9,14] REML: restricted maximum likelihood

Additionally, BCP resulted in a lower percentage of residual grafting materials compared to DBBM (MD=-8.42; 95% CI: -13.12 to -3.72; p<0.001; I^2^=85.71%; p<0.001) as shown in Figure 4.

**Figure 5:**
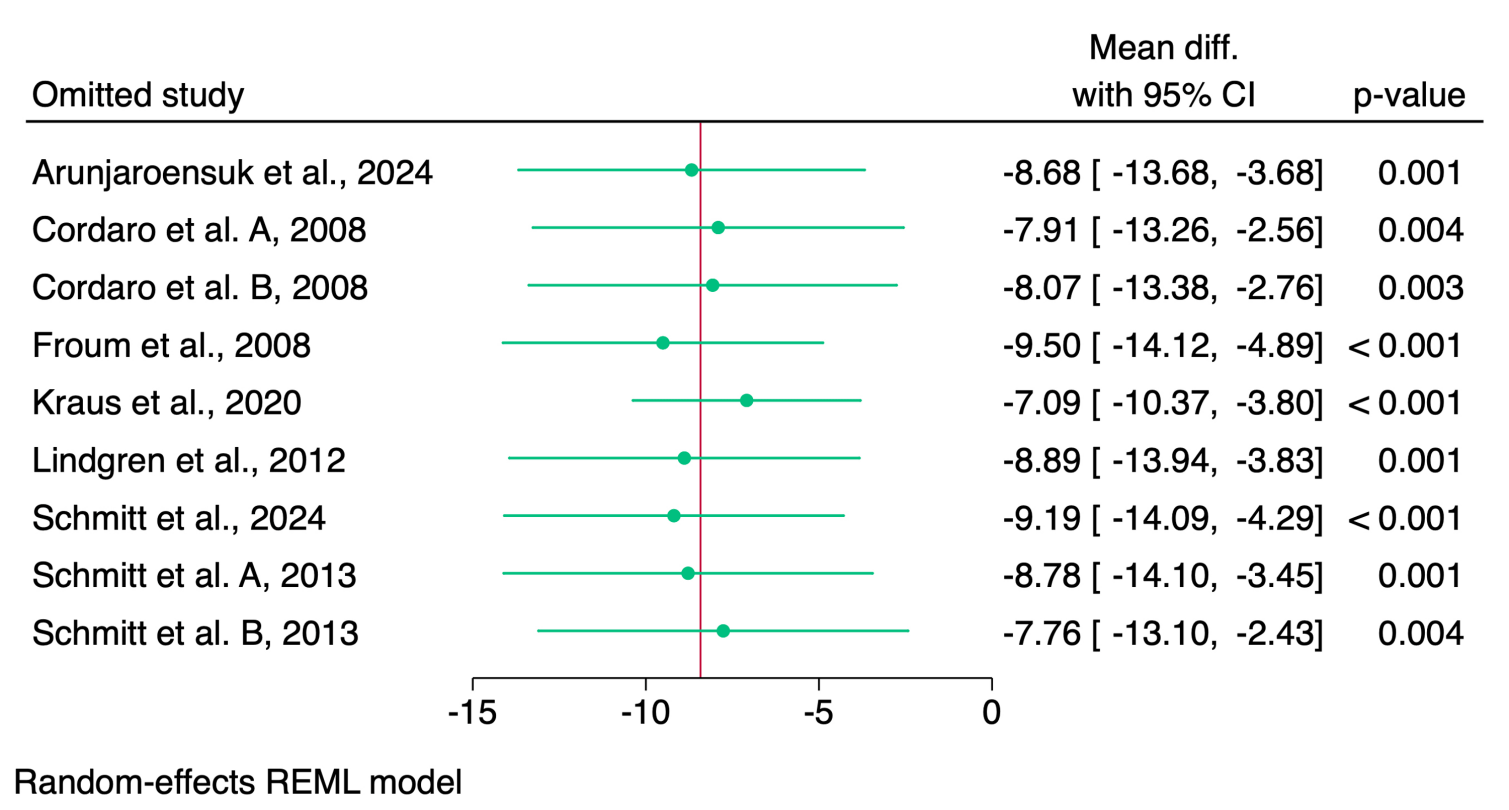
Leave-one-out sensitivity analysis of the percentage of residual grafting materials References: [1-9,14] REML: restricted maximum likelihood

Leave-one-out sensitivity analysis showed no significant impact of the individual studies on the pooled effect estimate, as shown in Figure 5.

**Figure 6:**
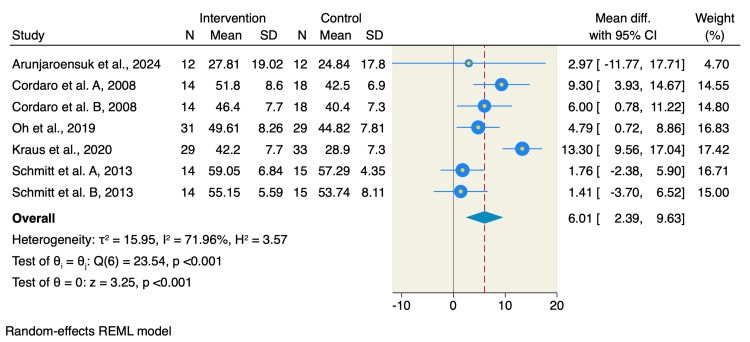
Forest plot of the percentage of soft tissue References: [1-9,14] REML: restricted maximum likelihood

Moreover, studies assessing the percentage change in the soft tissue showed that BCP resulted in an increase of the soft tissue compared to DBBM (MD=6.01; 95% CI: 2.39-9.63; p<0.001; I^2^=71.96%; p<0.001) as shown in Figure 6.

**Figure 7:**
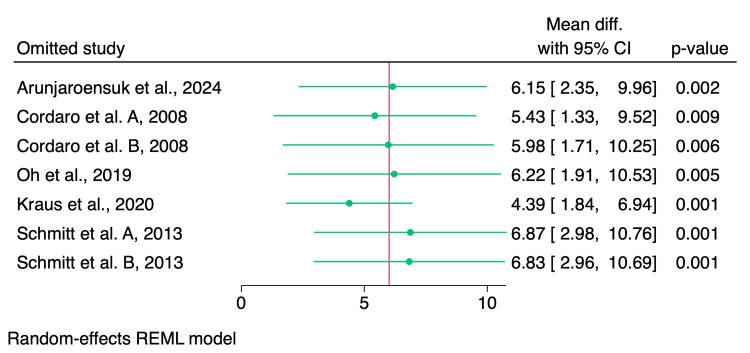
Leave-one-out sensitivity analysis of the percentage of soft tissue References: [1-9,14] REML: restricted maximum likelihood

Leave-one-out sensitivity analysis showed no significant impact of the individual studies on the pooled effect estimate, as shown in Figure 7.

Discussion

The current systematic review and meta-analysis of 10 RCTs and 328 patients is the most comprehensive study to date to address the histological outcomes related to the BCP compared to DBBM in patients requiring sinus floor augmentation. Our study showed that BCP was favored to increase the formation of new bone, with less residual grafting materials and an associated increase in the soft tissue materials compared to DBBM.

Histomorphometric analysis serves as a key method for assessing the healing dynamics of bone graft substitutes, offering insight into their osteoconductive potential, graft maturation timeline, and the extent of new bone formation [15]. Healing duration varies depending on the graft material, typically ranging from four to nine months [16]. Although certain studies have suggested a direct correlation between the extent of vital bone formation and the duration of graft maturation up to 12 months [15], further research is warranted to validate this association, particularly given the longer healing periods often observed in sinuses augmented with BCP.

Our findings demonstrated greater new bone formation following the use of BCP compared to DBBM. Previous studies have shown that sinus augmentation with bone grafting materials promotes new bone formation; however, Iezzi et al. [17] emphasized the need to maintain a balance between graft resorption and new bone development. Despite this, the ideal timeframe for complete graft resorption and subsequent bone regeneration within native bone remains unclear. During the early stages of healing, new bone tends to form between synthetic particles rather than directly on their surfaces [18], which may lead to reduced adhesion and contact with the host bone when compared to xenografts [6].

In accordance with the current findings, Lindgren et al., in a three-year follow-up study, compared BCP with DBBM and reported a similar amount of newly formed bone with each material (29% and 32%, respectively), along with comparable implant survival rates (96.8%) [8]. Likewise, in a histological study, Lindgren et al. found that new bone formation and bone-to-implant contact around implants were similar when the sinus was augmented with either BCP or DBBM [19].

Regarding other calcium phosphate materials, Trombelli et al. conducted a randomized, double-blind study comparing β-tricalcium phosphate and DBBM for transcrestal sinus floor elevation. Each group included 19 patients. The findings revealed that the extent of sinus elevation and the height of the graft apical to the implant apex were comparable immediately postoperatively. Notably, significant graft remodeling was observed in the β-tricalcium phosphate group at six months. Both materials were associated with minimal complications and postoperative discomfort, leading the authors to conclude that β-tricalcium phosphate and DBBM are both suitable options for this procedure [20].

Moreover, our findings were aligned with the meta-analysis conducted by Wu and his colleagues assessing the histological outcomes of sinus augmentation for dental implants with calcium phosphate or deproteinized bovine bone, summarizing five studies and 110 patients. They reported a higher percentage of bone formation following BCP without a significant difference [21]. Our results showed a significant difference, which could be explained by the inclusion of more RCTs with larger sample sizes and the exclusion of observational studies.

Our study has several limitations that should be considered when interpreting the findings. First, the number of included patients and implants was relatively small, and substantial heterogeneity existed across the studies. Additionally, the duration of follow-up after implantation, which can influence outcomes, was not consistently reported or analyzed. The type of implant used, a factor known to affect bone regeneration, was also not accounted for in the analysis, nor was the specific surgical technique employed.

Moreover, although histological outcomes are commonly used as surrogate markers for clinical performance, their predictive value should be interpreted with caution. While histological parameters may correlate with clinical success, they do not guarantee it; conversely, a clinically successful outcome may not always be supported by favorable histological findings. Furthermore, data on initial maxillary sinus volume and the precise amount of graft material used were not consistently available across studies, information that could have provided insights into graft resorption rates and new bone formation.

## Conclusions

Our meta-analysis on 10 RCTs and 328 patients showed that BCP resulted in an increase in new bone formation and soft tissue with a decrease in the residual amount of bone grafted compared to DBBM. Further long-term RCTs with predefined protocols of the healing time and grafted materials are recommended to confirm the current findings.
